# Systematic analysis of the regulatory functions of microRNAs in chicken hepatic lipid metabolism

**DOI:** 10.1038/srep31766

**Published:** 2016-08-18

**Authors:** Hong Li, Zheng Ma, Lijuan Jia, Yanmin Li, Chunlin Xu, Taian Wang, Ruili Han, Ruirui Jiang, Zhuanjian Li, Guirong Sun, Xiangtao Kang, Xiaojun Liu

**Affiliations:** 1 College of Animal Science and Veterinary Medicine, Henan Agricultural University, Zhengzhou 450002, China; 2 Henan Innovative Engineering Research Center of Poultry Germplasm Resource, Zhengzhou 450002, China; 3 International Joint Research Laboratory for Poultry Breeding of Henan, Zhengzhou 450002, China

## Abstract

Laying performance is an important economic trait in hens, and this physiological process is largely influenced by the liver function. The livers of hens at 20- and 30-week-old stages were investigated using the next generation sequencing to identify the differences of microRNA expression profiles. Compared with the 20-week-old hens, 67 down- and 13 up-regulated microRNAs were verified to be significant differentially expressed (false discovery rate, FDR ≤ 0.05) (SDE) in the 30-week-old. We also identified 13 down- and 6 up-regulated novel differentially expressed (DE) microRNAs. miR-22-3p and miR-146b-5p, which exhibit critical roles in mammalian lipid metabolism, showed the most abundant expression and the highest fold-change, respectively. A total of 648 potential target genes of the SDE microRNAs were identified through an integrated analysis of microRNAs and the DE genes obtained in previous RNA-sequencing, including *FADS1*, *FADS2, ELOVL6* and *ACSL5*, which are critical lipid metabolism-related regulators. Bioinformatic analyses revealed that target genes were mainly enriched in lipid-related metabolism processes. This work provides the first study of the expression patterns of hepatic microRNAs between 20- and 30-week old hens. The findings may serve as a fundamental resource for understanding the detailed functions of microRNAs in the molecular regulatory systems of lipid metabolism.

Laying eggs is the most important economic trait in egg-laying hens, and this physiological process is largely influenced by chicken liver function. Numerous studies have demonstrated that most of the genes and their products involved in poultry hepatic lipid metabolism are similar to those of mammalian species; however, the functions of a number of these genes and their products in poultry are largely different from their counterparts in mammals^1^,^2^,^3^,^4^. Moreover, a previous study indicated that poultry species may have lost some of the genomic genes involved in lipid metabolism (e.g., resistin, *TNFα*, *PAI-1*) during the evolutionary process compared with mammals^5^. Although RNA-seq studies of chicken liver have reported differentially expressed (DE) genes acting mainly on the regulation of lipid metabolism between pre- and peak-laying stages^6^, the roles of molecular regulators (e.g., microRNAs, lncRNAs) on poultry hepatic lipid metabolism require further investigation.

MicroRNAs (miRNAs) are a class of endogenous, evolutionarily conserved small non-coding RNAs that are approximately 22 nucleotides (nt) in length^7^. With a typical hairpin loop structure^8^, miRNAs are transcribed initially from primary transcripts that are encoded either in intergenic regions or within overlapping genes (non-protein coding or coding) as primary miRNAs. In general, miRNAs interact with mRNAs to perform their functions. For example, in mammalian species, it has been estimated that only 1–5% of genomic transcripts code miRNAs, but up to 60% of the genes are directly or indirectly regulated by miRNAs. It has been argued that one miRNA can regulate the expression of hundreds of mRNAs, and the expression of one mRNA could be regulated simultaneously by hundreds of miRNAs^9^. In other words, miRNAs can play critical roles through constructing networks of sophisticated regulatory control systems in organisms^10^.

Increasing numbers of studies have demonstrated that miRNAs serve as important regulators in hepatic metabolism. For example, the mammalian liver-specific miR-122, which is expressed abundantly in liver, can modulate liver protein metabolism by targeting the regulation of cationic amino acid transporter 1^11^. MiR-122 can also regulate hepatic fatty acids and cholesterol synthesis by repressing the expression of genes involved in cholesterol biosynthesis^12^,^13^. Furthermore, miR-33 can function in liver metabolism by regulating cholesterol efflux and high-density lipoprotein metabolism via targeting the ATP-binding cassette sub-family A member 1 and ATP-binding cassette sub-family G member 1^14^. MiR-33 can also inhibit the translation of multiple transcripts encoding proteins involved in fatty acid β-oxidation, which could therefore reduce fatty acid degradation^15^. MiRNA-dependent post-transcriptional gene silencing is now recognized as an important element of lipid metabolism^12^,^16^,^17^.

The physiological process of poultry egg-laying requires lipid-related metabolism, which could be highly related with the hepatic miRNA-mediated lipid metabolism. Given that 20- and 30-week-old layer hens have reached their sexual maturation and that the most significant physiological difference between them is whether or not they lay eggs, we hypothesized that the expression of hepatic miRNAs in peak-laying hens would differ from that of the pre-laying. Thus, we adopted the miRNA-seq technology to investigate the expression of hepatic miRNAs in 20- and 30-week-old layer hens. An integrated analysis of significant differentially expressed (SDE) miRNAs and DE hepatic genes retrieved from a previous publication^6^ was performed to elucidate the regulatory patterns of miRNAs and their network with putative target genes. Investigating the molecular regulatory mechanism of chicken hepatic lipid metabolism could not only contribute to deeper understanding of its regulatory system but also be of benefit in efforts to enhance the egg-laying performance of poultry.

## Results

### Sequence analyses of the sRNAs

A summary of the matching count data for the non-coding RNAs reads and small RNAs (sRNAs) in the liver of 20- (L20) and 30-week (L30) libraries is presented in Tables 1 and 2, respectively. A total of 57,648,491 and 62,999,617 raw reads were obtained from the L30 and L20 libraries, respectively. After removing contaminant reads, we obtained 54,285,329 (L30) and 61,393,581 (L20) clean reads that were used for following analyses. Among the clean reads, an average of 13,747,104 sequences from the L20 libraries and 8,645,866 sequences from the L30 libraries mapped perfectly to the chicken genome sequence (Table 1). The average numbers of sRNAs reads were 1,470,389 and 2,186,271 in the L20 and L30 libraries, respectively. The average counts of miRNAs that were annotated in miRBase 21.0 (*Gallus gallus*) were 32,782 (L20) and 22,931 (L30), and the average numbers of known miRNAs were 408 and 383 in the L20 and L30 libraries, respectively (Table 2).

**Figure 1:**
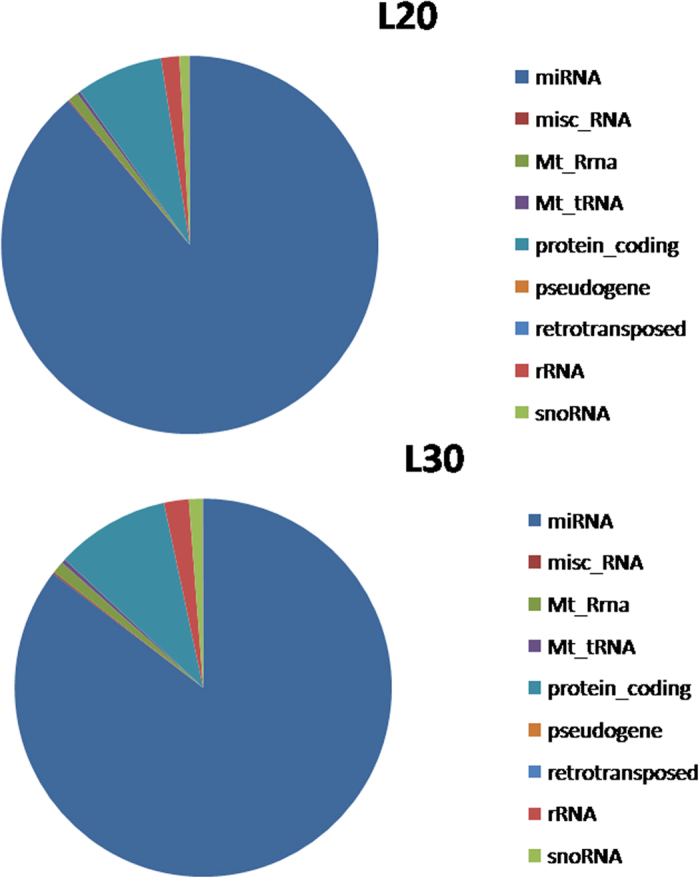
Distribution of different sRNA classes in the miRNA-seq data. L20, library prepared from the livers of 20-week-old chickens; L30, library prepared from the livers of 30-week-old chickens.

**Figure 2:**
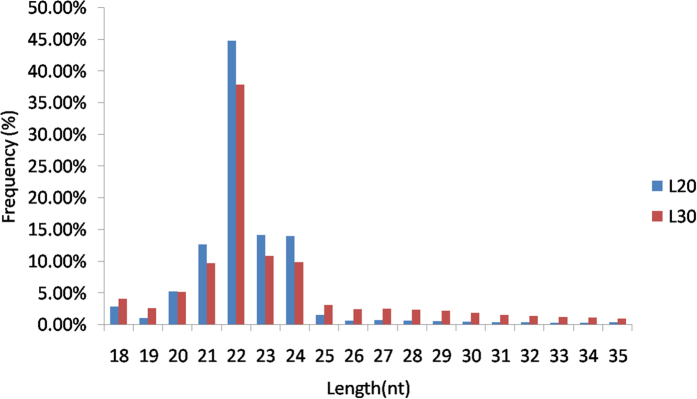
Length distribution of miRNA sequences from the livers of 20- and 30-week-old chickens.

The sequenced sRNAs were mapped to a number of public databases (see Materials and Methods for details), and classified as rRNAs, known miRNAs (miRNAs in miRBase 21.0), misc_RNAs, Mt_rRNAs, Mt_tRNAs, protein-coding mRNAs, pseudogenes, retrotransposons, and snoRNAs (Fig. 1). More than 80% of the sRNA sequences were miRNAs. The majority of the sRNA reads were 21–24 nt in length in both groups (Fig. 2). The 22-nt sRNAs were the most abundant, accounting for over 40% of the total sequence reads. This group was followed by the 21, 23, and 24 nt sRNAs. These values fall into the typical range of miRNAs for Dicer-derived products. For each library, the sequence depth was greater than 10 M, thus reaching saturation (Supplementary Fig. S1). In addition, novel miRNAs with a size ranging from 21 to 23 nt were identified, and the 5′ ends of most of them were comprised of uridine (U).

### Analyses of chicken hepatic miRNAs

**Figure 3:**
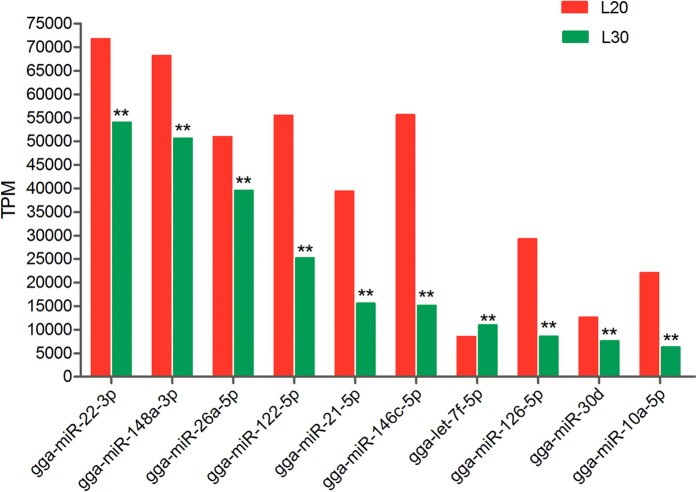
Top ten most abundantly expressed miRNAs in the livers of 20- and 30-week-old chickens. **FDR ≤ 0.05.

To identify the SDE miRNAs that may play important regulatory roles in chicken liver, we compared the expression patterns of hepatic miRNAs at 20 and 30 weeks. Of the 996 *Gallus gallus* mature miRNAs in miRBase 21.0, 565 were represented in the L20 and L30 libraries (Supplementary Table S1). All of the top 10 most abundant miRNAs were significantly expressed (FDR < 0.05) in L30 chicken liver libraries (Fig. 3). Nine of them were down-regulated in L30 compared with L20; the exception was let-7f-5p, which was up-regulated (1.29-fold). MiR-22-3p (−1.33-fold) exhibited the highest expression levels in both libraries, followed by miR-148a-3p (−1.35-fold), whereas miR-146c-5p (−3.69-fold) exhibited the maximum fold-change.

Among the 71 novel miRNAs, 60 were found in L30, and 67 were found in L20. In addition, 56 were shared between the libraries. Compared with L20, 19 DE novel miRNAs (*P* ≤ 0.05), including 13 down- and 6 up-regulated miRNAs, were identified in L30. The novel miRNA gga01 was down-regulated in L30 with the highest fold-change (−17.97-fold), and some of the novel miRNAs were detected in only one of the libraries (Supplementary Table S2); however, the expression levels of the majority of the novel miRNAs were relatively low, except gga56. Among the 565 known miRNAs, 80 were SDE (FDR ≤ 0.05) between the libraries; 67 were down-regulated, and 13 were up-regulated, using L20 as the baseline (Supplementary Table S3). The SDE miRNAs with high fold-changes were all down-regulated in L30, including miR-146b-5p (−8.50-fold), miR-24-3p (−7.39-fold), miR-146a-5p (−5.96-fold), miR-221-5p (−5.85-fold), miR-7b (−5.35-fold), miR-147 (−5.11-fold), miR-20-5p (−4.59-fold), and miR-140b-5p (−4.57-fold). Seven conserved families all were DE with *P* ≤ 0.05, including let-7 (let-7a, -7b, -7c,-7f, -7g, -7i, -7j, and -7k), miR-130 (miR-130a, and -130b), miR-146 (miR-146a, -146b, and -146c), miR-15 (miR-15a, -15b, and -15c), miR-181 (miR-181a and -181b), miR-29 (miR-29a, -29b and -29c), and miR-30 (miR-30a, -30b, -30c, -30d, and -30e). All members of the miR-15, miR-181, and miR-29 families were down-regulated in L30 compared with L20, whereas the other families included members that were either up- or down-regulated.

### qRT-PCR validation of the sequencing data

**Figure 4:**
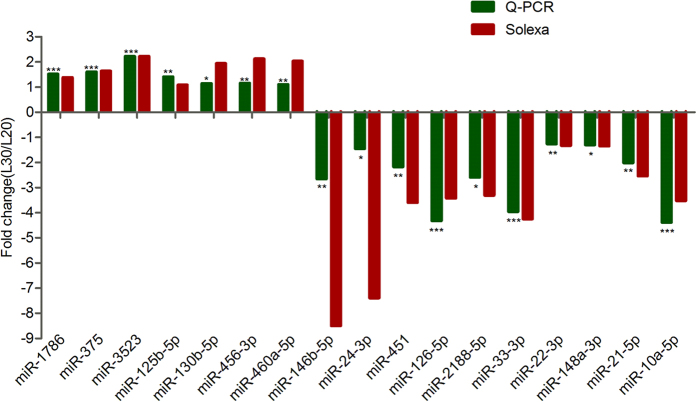
QRT-PCR verification of differentially expressed miRNAs. *P ≤ 0.05; **P ≤ 0.01; ***P ≤ 0.001. Fold-change > 0 indicates up-regulation; fold-change < 0 indicates down-regulation.

To validate the reliability of sequence data obtained from high-throughput sequencing, we performed stem-loop qRT-PCR. Seventeen miRNAs with different expression levels were selected randomly; one was not significantly expressed (miR-1786), the others are sixteen SDE miRNAs including six up-regulated (miR-375, -3523, -125b-5p, -130b-5p, -456-3p, and -460a-5p), and ten down-regulated miRNAs (miR-146b-5p, -24-3p, -451, -126-5p, -2188-5p, -33-3p, -22-3p, -148a-3p, -21-5p, and -10a-5p) (Fig. 4). The results showed that the expressions of the selected miRNAs were significant and consistent with the miRNA-sequencing results (Fig. 4).

### Integrated analyses and functional annotation

To identify the candidate biological processes in which the identified miRNAs may be involved, we integrated the target genes of the SDE miRNAs and DE genes obtained from the chicken liver transcriptome data^6^.

**Figure 5:**
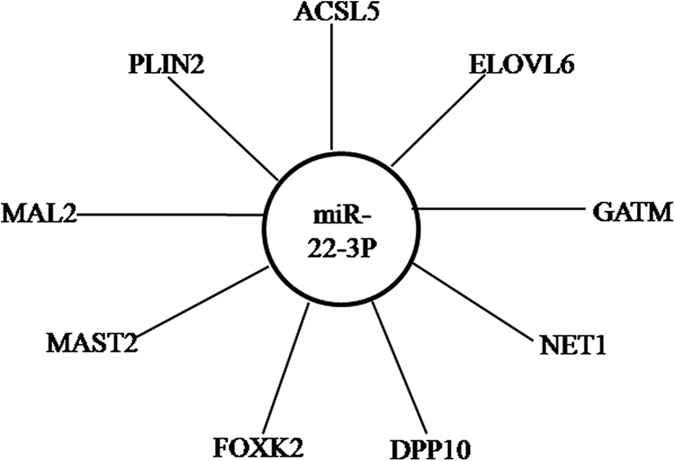
The putative target genes of miR-22-3p.

A total of 648 DE genes were potentially targeted by the SDE miRNAs. With the highest abundant expression, down-regulated miR-22-3p was predicted to target nine genes (Fig. 5), including ELOVL fatty acid elongase 6 (*ELOVL6*), long-chain acyl-CoA synthetases (*ACSL5*) and perilipin 2 (*PLIN2*), which are the key regulatory factors in lipid metabolism. MiR-101-3p (−2.1-fold) and miR-15c-5p (−1.4-fold) had the most target genes followed by miR-15a, miR-16-5p, miR-214, miR-16c-5p, and miR-181b-5p (Supplementary Table S4), and these miRNAs were all down-regulated in L30 compared with L20.

**Figure 6:**
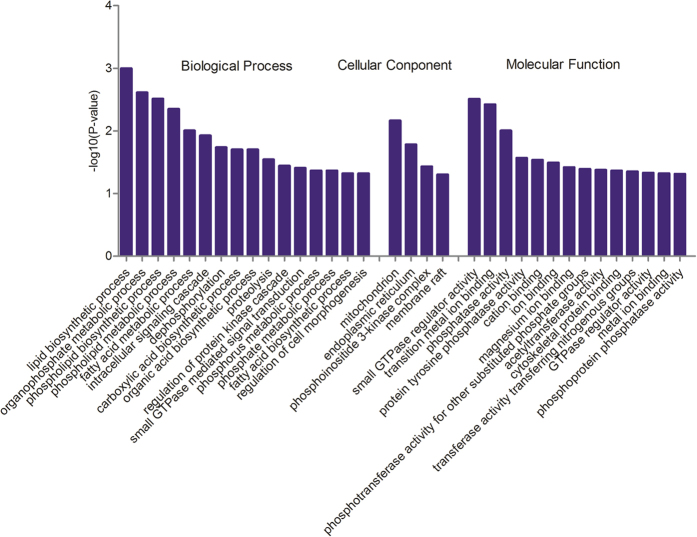
Enriched GO terms of differentially expressed genes targeted by the significant differentially expressed miRNAs. Only the significantly enriched (*P* ≤ 0.05) GO terms in the biological process, cellular component, and molecular function categories are presented.

All of the target genes were assigned to GO terms using DAVID. Sixteen significantly enriched terms in the biological process category (*P* ≤ 0.05) were identified. In particular, some target genes were identified through the annotation as being involved in lipid biosynthetic process, organophosphate metabolic process, phospholipid biosynthetic and phospholipid metabolic process, and fatty acid metabolic and biosynthetic process, all of which are closely associated with the regulation of lipid metabolism (Fig. 6). Mitochondrion, endoplasmic reticulum, phosphoinositide 3-kinase complex, and membrane raft were significant enrichment terms in the cellular component category. In the molecular function category, small GTPase regulator activity, phosphatase activity, protein tyrosine phosphatase activity, and phosphotransferase activity for other substituted phosphate groups were also significantly enriched. In particular, five pathways, including steroid biosynthesis, glycerophospholipid metabolism, biosynthesis of unsaturated fatty acids pathways, pantothenate and CoA biosynthesis, and PPAR signalling pathway, which are relevant to lipid metabolism, were significantly enriched (Table 3). Furthermore, other pathways concerned with pantothenate and CoA biosynthesis, amino acid metabolism, fructose and mannose metabolism, and drug metabolism may be actively regulated by miRNAs in chicken liver.

**Figure 7:**
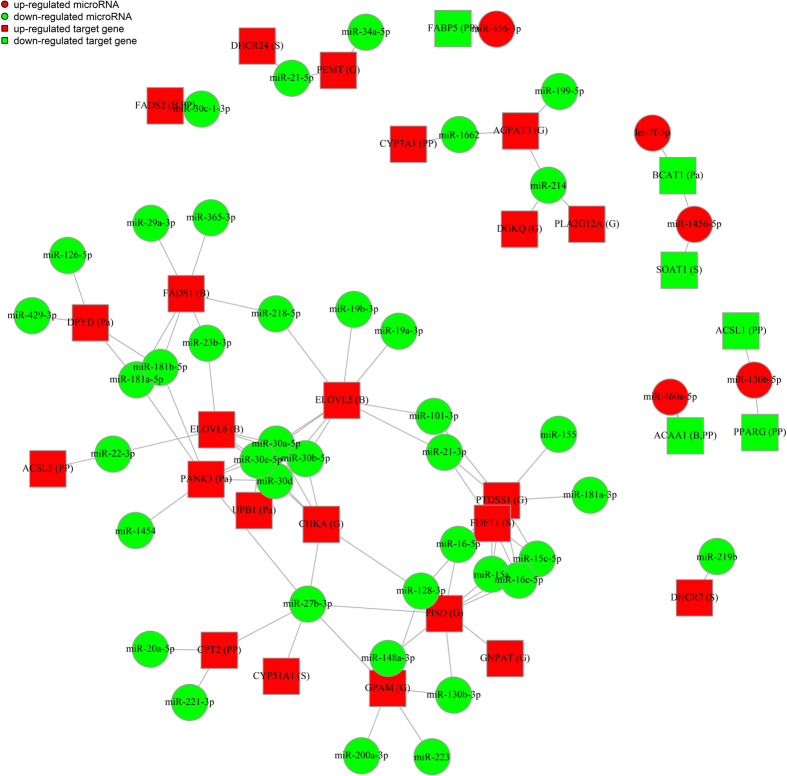
Integrated analysis of miRNAs and their target genes enriched in significant pathways. Circle indicates miRNA, and square indicates target gene. Green indicates down-regulation, and red indicates up-regulation. The letter in the bracket after the target gene indicates the abbreviation of the related pathways as follows: Pa-Pantothenate and CoA biosynthesis, B-Biosynthesis of unsaturated fatty acids, G-Glycerophospholipid metabolism, PP-PPAR signalling pathway, and S-Steroid biosynthesis.

To better understand the interactions, we visualized the integrated miRNAs-mRNAs networking among the SDE miRNAs and their target genes significantly enriched in the above-mentioned five pathways (Fig. 7). The results indicated that nine genes, which were enriched in the glycerophospholipid metabolism pathway, were specifically targeted by down-regulated miRNAs, and five of these nine genes were targeted by miR-128-3p. The fatty acid desaturase *FADS2*, which catalyses the initial desaturation step in the synthesis of long chain polyunsaturated fatty acids, was included in both the PPAR signalling pathway and the biosynthesis of unsaturated fatty acids pathway and was targeted by the down-regulated miRNA miR-30c-1-3p. In contrast, *FADS1*, which encodes the fatty acid desaturase 1 that is involved in the biosynthesis of unsaturated fatty acids, was targeted by six down-regulated miRNAs, including miR-365-3p, -218-5p, -181a-5p, -181b-5p, -29a-3p, and -23b-3p. The genes encoding phosphatidylserine synthase 1 (*PTDSS1*) and fatty acid elongase 5 (*ELOVL5*) are involved in the glycerophospholipid metabolism and biosynthesis of unsaturated fatty acids pathways, respectively. These two genes were targeted by the highest numbers of miRNAs, including miR-101-3p and miR-21-3p. Both the sterol O-acyltransferase 1 (*SOAT1*) and branched chain amino-acid transaminase 1 (*BCAT1*) genes were targeted by miR-1456-5p. *CYP7A1*, which is the target of miR-1662 and encodes a cytochrome P450 that catalyzes the rate limiting step of the conversion of cholesterol to bile acids^18^, was included in the PPAR signalling pathway. In addition, 1-acylglycerol-3-phosphate O-acyltransferase 3 (*AGPAT3*), another target of miR-1662 that encodes an O-acyltransferase, was associated with glycerophospholipid metabolism. In addition, the putative target genes of miR-30 family members (miR-30a, -30b, -30d, -30e, and -30c) encode proteins involved in glycerophospholipid metabolism, biosynthesis of unsaturated fatty acids, pantothenate and CoA biosynthesis, and PPAR signalling pathway.

## Discussion

Eggs are important contributors and rich sources of lipids, such as cholesterol and phospholipids^19^. In chicken, more than 90% of the *de novo* synthesis of fatty acids occurs in liver^20^,^21^,^22^; therefore, liver metabolism has a critical effect on the performance of chicken egg-laying. Although the mechanisms of hepatic lipid metabolism have been studied extensively in chicken, the underlying molecular regulatory mechanisms and the roles of miRNAs in the process remain to be fully determined. In the current study, we adopted miRNA-seq technology to investigate the expression profile of hepatic miRNAs related to the mechanism of lipid metabolism in chickens at two different physiological stages (20 and 30 weeks). To our knowledge, this is the first miRNA-seq profiling study of chicken liver to explore the role of hepatic miRNAs and their putative DE target genes in lipid metabolism.

In total, 565 known and 71 potential novel miRNAs were identified in this study. Among them, miR-22-3p, as the most highly abundant SDE miRNA, was predicted to target *ACSL5*, *ELOVL6* and *PLIN2,* all of which are involved in lipid metabolism. *ACSL5* plays an important role in partitioning fatty acids toward triglyceride^23^, and its suppression could result in a decrease in the formation of fatty acid-induced lipid droplets^24^. In contrast, the deletion of *ELOVL6*^25^ and *PLIN2*^26^ suppress the accumulation of hepatic triglyceride. Furthermore, miR-22-3p is critical in the fatty liver development in mice by modulating target genes in a way that can lead to an increase in lipid accumulation when tested in human hepatoma (HepG2) cells^27^,^28^,^29^,^30^,^31^, implying that it could be an important regulator in promoting chicken hepatic lipid synthesis. In addition, some other miRNAs, such as miR-148a, miR-122, miR-21-5p, Let-7f-5p, miR-26a-5p, miR-126-5p, miR-30d, and miR-10a-5p, were also highly abundant in chicken liver. A previous study demonstrated that miR-148a, miR-122, and miR-21-5p were the most abundant miRNAs in porcine liver^32^. The inhibition of miR-122 expression could reduce plasma cholesterol levels and decrease the synthesis rate of hepatic fatty acids and cholesterol in the mouse liver^12^.

Both 20- and 30-week-old layer hens have reached sexual maturation, and the most significant physiological difference between the groups is whether they lay eggs. Therefore, the changes in the miRNA/mRNA expression profiles between 20- and 30-week-old layer hens could mainly be attribute to egg production. However, we were unable to completely exclude the possibilities that some of the changes may be related to normal development and/or environmental variations despite being strictly controlled. In fact, some significantly changed miRNAs were previously reported to be related to growth and development in other species. For example, miR-126, -30d and -10a are relevant to porcine muscle development^33^, and miR-148a mediates myogenic differentiation through targeting ROCK1^34^. These conserved miRNAs may play the similar roles in chicken though we don’t know yet.

Among the identified miRNAs in this study, 80 SDE known and 19 DE novel miRNAs were detected at the 30-week stage compared with the 20-week stage, and four of these novel miRNAs were exclusively observed at the 30-week stage. On the one hand, many of these known SDE miRNAs are related to hepatic lipid metabolisms. For instance, miR-146b-5p (also known as miR-146b) is located in an intergenic region (22,683,802–22,683,906) on chicken chromosome 6 and is found in most vertebrate species (including mammals). MiR-146b-5p is highly conserved, exhibiting a −8.50-fold change at 30 weeks compared with 20 weeks. This change could directly influence lipid metabolism in chicken liver because a previous study demonstrated that it could regulate adipogenesis^35^. Consistent with the fold-change in miR-146b-5p, miR-24-3b was down-regulated (−7.39-fold) in 30-week-old chickens. MiR-24-3b may respond to a dramatic increase in lipid accumulation in 30-week old chicken liver and could therefore be down-regulated to maintain hepatic lipid homeostasis. A previous study demonstrated that the over-expression of miR-24 could indirectly promote hepatic lipid accumulation and hyperlipidaemia and that its knock down could lead to lipogenesis^36^. On the other hand, the novel miRNAs all had quite low expression levels, with the exception of gga56, which was relatively highly expressed. The novel miRNA gga01 was notably down-regulated in the peak-laying stage with the highest fold-change (−17.97-fold). Though it could be challenging to identify the functions of these novel miRNAs, investigations regarding the underlying mechanisms are warranted.

Hepatic miRNAs could also play important roles in regulating various hepatic functions^27^. A previous study demonstrated that miRNAs are associated with energy metabolism through the modulation of glucose and lipid homeostasis via binding to their target genes^37^. For example, miR-33a and miR-33b jointly with their target *SREBP* encoding gene are important transcriptional regulators of genes involved in lipogenesis^38^ and regulators of cholesterol homeostasis and fatty acid metabolism^39^. Another example is miR-122, which is a liver-specific miRNA that regulates hepatic fatty acid oxidation and fatty acid and cholesterol synthesis rates^12^,^40^.

Furthermore, the 648 predicted target genes of the SDE miRNAs were annotated with GO terms and subjected to a GO enrichment analysis. This demonstrated that some of these genes were mainly involved in the regulation of lipid metabolism. Steroid biosynthesis, glycerophospholipid metabolism, biosynthesis of unsaturated fatty acids, and the PPAR signalling pathway were the significantly enriched lipid-related metabolic processes.

The miRNAs-mRNAs network revealed the interaction among the molecules. *ELOVL6* and *ACSL5* proteins, the target genes of miR-22-3p, are involved in the biosynthesis of unsaturated fatty acids and PPAR signalling pathways, respectively. These results indicate that miR-22-3p may be involved in lipid accumulation by binding to target genes involved in chicken hepatic fatty acid metabolism. *CYP7A1*, which is associated with the conversion of cholesterol to bile acids and cholesterol homeostasis^18^,^41^, and *AGPAT3* were both predicted to be targeted by miR-1662. A variant of *AGPAT3* is one of the determinants in circulating glycerophospholipids and sphingolipid^42^, and the AGPAT3 protein participates in the incorporation of docosahexaenoic acid (DHA) into phospholipids^43^. Five of the potential targets of miR-128-3p were present in the glycerophospholipid metabolism biological pathway. Members of the miR-30 family are important positive regulators of adipocyte differentiation in a human adipose tissue-derived stem cell model^44^. *FADS1* and *FADS2* play important catalytic roles in the critical steps of the long chain polyunsaturated fatty acids biosynthesis process^45^,^46^. In this study, *FADS1* was targeted by miR-365-3p, miR-218-5p, miR-181a-5p, miR-181b-5p, miR-29a-3p, and miR-23b-3p, whereas *FADS2* was targeted by miR-30c-1-3p. Moreover, *SOAT1* (also known as acyl-Coenzyme A: cholesterol acyltransferase 1, ACAT), the putative target gene of miR-1456-5p, synthesizes cholesterol fatty acid esters using fatty acids released from membrane phospholipids^47^. However, the detailed function of these miRNAs on their target genes in the network needs further investigation to clarify the regulation of chicken hepatic lipid metabolism.

In summary, the findings in this study are consistent with our hypothesis that the expression profile of miRNAs differs between 20- and 30-week-old hens. Some of the identified SDE miRNAs could serve as critical regulators in the networking of key pathways (e.g., steroid biosynthesis, biosynthesis of unsaturated fatty acids pathways) involved in lipid-related metabolism, Moreover, the findings also indicated a direct role of miRNA-mediated post-transcriptional regulation in chicken hepatic lipid metabolism during the peak-laying stage. This work provided the first study of the expression profile of hepatic miRNAs between 20- and 30-week-old members of an important economic model species, and it may serve as a fundamental resource for further studies on this topic and other related fields.

## Methods

### Ethics Statement

All animal experiments were performed in accordance with the protocol approved by the Institutional Animal Care and Use Committee (IACUC) of Henan Agricultural University^6^.

### Sample collection and RNA extraction

The experimental animals used in this study were a Chinese domestic breed known as Lushi green-shell laying hens. All chickens were raised in cages under the same environment with *ad libitum* water and food. Healthy individuals were sampled randomly and then slaughtered at the stage of 20 weeks old (pre-laying) and 30 weeks old (peak-laying). Liver tissue was immediately collected, snap-frozen in liquid nitrogen, and stored at −80 °C. The small RNA (sRNA) used for sequencing was extracted using a mirVana™ miRNA Isolation Kit (Ambion, Austin, TX, USA) according to the manufacturer’s instructions. The quality and quantity of the total RNA were assessed using an Agilent 2100 (Agilent Technologies, Santa Clara, CA, USA) Bioanalyzer system. sRNA with 28S/18S ratios ranging from 1.8 to 2.0 and RNA integrity number values of between 8.0 and 10.0 was selected for further analysis. The RNA samples were stored at −80 °C until further use.

### Small RNA library construction and RNA-sequencing

Six sRNA libraries of chicken livers were constructed from the 20-week (L20-1, L20-2, and L20-3) and 30-week (L30-1, L30-2, and L30-3) chicken livers and prepared for sequencing analysis. Briefly, 3′ and 5′ RNA adapters were ligated successively to the total RNA with T4 RNA ligase. Subsequently, cDNA was obtained by reverse-transcribed PCR from the ligated RNAs. Then, the cDNA was amplified by PCR. Amplification products with appropriate lengths were purified from agarose gel to construct the sequence libraries, which were sequenced using a single-read 1 ×36 nt multiplex procedure on a Genome Analyzer IIx (Illumina, Inc., San Diego, CA, USA) following the manufacturer’s instruction.

### Data analyses

The raw reads were filtered using the Fastx (fastx_toolkit-0.0.13.2) pre-processing tool to remove adaptor sequences, low-quality reads (including reads with unknown bases N), reads smaller than 18 nt, and reads with a base quality less than 10. The clean reads from each sample were aligned to sequences in the miRBase database 21.0 using CLC Genomics Workbench 5.5 commercial software (CLC bio, Aarhus, Denmark). Un-mapped reads were annotated and classified by searching against the non-coding RNA sequences (piRNA, tRNA, snoRNA, rRNA, and snRNA) in the GenBank (http://www.ncbi.nlm.nih.gov/) and Ensembl ncRNA databases (http://asia.ensembl.org/index.html) as well as the RNA families in Rfam (http://rfam.sanger.ac.uk/) and the Piwi-interacting RNAs in piRNA Bank (http://pirnabank.ibab.ac.in/). The maximum number of mismatches allowed was two bases, and a two-base shortening or extension at both ends of the sequence was allowed during the alignment. The miRCat tool in the sRNA tool kit^48^ was used to predict novel miRNAs. The remaining non-annotated sRNA sequences were aligned against the chicken genome sequence^49^, and genomic sequences containing the sRNA were used to predict hairpin structures with the Mfold program (http://mfold.rna.albany.edu). Only sequences exhibiting a typical stem-loop hairpin structure that were expressed at least in three samples were considered to be candidate novel miRNAs. When all of the annotation steps were completed, the sequencing libraries were subjected to size distribution and saturation analyses.

### Differential expression analyses

The expression levels of the annotated miRNAs were estimated from the Illumina sequencing data based on transcripts per million clean reads (TPM)^50^. The calculated TPM values were used to compare miRNA expression levels between the two physiological stages (20 and 30 weeks). The fold-change for each miRNA between the two stages was calculated as L30/L20 using the TPM values. The DEGseq R package^51^ was used to analyze the differentially expressed miRNAs. *P*-values were determined by the Fisher test and the FDR was used to adjust the threshold of the *P*-value for multiple tests. MiRNAs with FDR ≤ 0.05 were identified as SDE miRNAs^52^.

### Quantitative real-time PCR (qRT-PCR)

The expression levels of some randomly selected miRNAs were validated by qRT-PCR. The RNA used for the PCR was reversely transcribed using a cDNA Synthesis kit (TaKaRa, Dalian, China) according to the manufacturer’s instruction. The relative expression levels of the miRNAs were quantified using the SYBR Green method in a LightCycler^®^ 96 instrument (Roche Applied Science). Chicken small nuclear RNA U6 was used as the internal control. The loop primers used for the qRT-PCR were ordered from Shanghai GenePharma Co., Ltd (Shanghai, China). The PCR amplification process was as follows: 95 °C for 3 min; 40 cycles of 95 °C for 12 sec, 61 °C for 40 sec, 72 °C for 30 sec; 10 min of extension at 72 °C. All the reactions were run in three replications, and the relative expression levels were calculated using the 2^−ΔΔct^ method^53^. The significance of the expression levels were determined by a t-test (unpaired, two-tailed) using Graphpad Prism 5 (Graphpad Software, San Diego, CA). *P*-values ≤ 0.05 were considered statistically significant^17^.

### Prediction and functional analyses of miRNA target genes

Potential target genes of the identified known chicken SDE miRNAs were predicted using the computational algorithm miRanda with the principle of TargetScan^54^,^55^. For each SDE miRNA that was up-regulated in the 30-week group, the potential target genes were predicted among the down-regulated DE genes in the RNA-seq data of the 30-week group^56^. Similarly, the potential DE target genes of the down-regulated SDE miRNAs in the 30-week group were predicted from the up-regulated DE genes.

All the potential DE target genes of the SDE miRNAs were used in the bioinformatics analysis. Functional annotation analysis was performed using DAVID web-based tools^57^ to identify enriched Kyoto Encyclopedia of Genes and Genomes (KEGG) pathways^58^ and gene ontology (GO) terms, group functionally related genes, and cluster annotation terms for large gene lists^59^. Only GO terms and pathways with *P*-values ≤ 0.05 were included in the analysis. The network interactions between miRNAs and their related target genes were conducted using the “igraph” package in R (version 3.2.2)^60^.

### Accession numbers

All the Illumina miRNA-seq data sets supporting the results of this article have been submitted to the National Center for Biotechnology Information (NCBI) Gene Expression Omnibus (GEO) under accession number GSE74242.

## Additional Information

**How to cite this article**: Li, H. *et al*. Systematic analysis of the regulatory functions of microRNAs in chicken hepatic lipid metabolism. *Sci. Rep.* **6**, 31766; doi: 10.1038/srep31766 (2016).

## Supplementary Material

Supplementary Information

## Figures and Tables

**Table 1:** Descriptive summary of non-coding RNAs reads.

ID^a^	Raw Reads	Clean reads	Percent (%)^b^	Annotated reads	Perfect matches^c^	Reads Annotatedin in miRBase21.0 (*Gallus gallus*)	Reads Perfect matches in miRBase21.0 (*Gallus gallus*)
L20-1	20,905,809	20,300,052	97.1%	17,392,373	14,041,252	12,170,502	10,089,552
L20-2	27,180,553	26,552,542	97.7%	22,368,399	17,966,690	15,306,101	12,614,600
L20-3	14,913,255	14,540,987	97.5%	11,482,606	9,233,371	7,377,736	6,131,583
L30-1	20,677,403	19,561,788	94.6%	14,870,023	11,687,333	9,079,113	7,804,188
L30-2	15,124,850	13,450,426	88.9%	7,916,715	6,298,661	4,594,483	4,070,144
L30-3	21,846,238	21,273,115	97.4%	10,503,394	7,951,605	3,972,398	3,431,075

^a^L20, liver samples from 20-week-old layer hens; L30, liver samples from 30-week-old layer hens.

^b^Percent, clean reads/raw reads.

^c^Perfect matches, reads that matched the reference genome completely.

**Table 2:** Summary of small RNAs matching counts.

ID	Small RNA count	Annotated small RNA count	Annotated In miRBase21.0 (*Gallus gallus*)	Percent (%)	Sequence found^a^
L20-1	1,091,609	233,388	34,233	14.7	400
L20-2	1,716,360	326,430	37,501	11.5	430
L20-3	1,603,197	238,233	26,613	11.2	394
L30-1	1,299,686	273,917	29,070	10.6	382
L30-2	1,379,829	314,904	20,138	6.4	352
L30-3	3,879,299	313,723	19,584	6.2	417

^a^Sequence found, the miRNA sequence existed in miRbase 21.0.

**Table 3:** Summary of pathways associated with DE genes targeted by SDE miRNAs.

ID	Term	*P-value*	Genes
gga00100	Steroid biosynthesis	0.004797	*SOAT1, CYP51A1, DHCR7, DHCR24, FDFT1*
gga00564	Glycerophospholipid metabolism	0.013502	*CHKA, DGKQ, PLA2G12A, PEMT, GNPAT, PISD, PTDSS1, GPAM, AGPAT3*
gga01040	Biosynthesis of unsaturated fatty acids	0.018062	*ELOVL5, FADS1, FADS2, ELOVL6, ACAA1*
gga00770	Pantothenate and CoA biosynthesis	0.020411	*BCAT1, PANK3, UPB1, DPYD*
gga03320	PPAR signaling pathway	0.04317	*CPT2, ACSL1, CYP7A1, PPARG, FADS2, FABP5, ACAA1, ACSL5*
