# Supplementary material for: Systematic analysis of the regulatory functions of microRNAs in chicken hepatic lipid metabolism
Source: Sci Rep. 2016 Aug 18;6:31766. doi: 10.1038/srep31766 (PMC4989143; doi:10.1038/srep31766)

Supplementary information file

Systematic analysis of the regulatory functions of microRNAs in chicken hepatic lipid metabolism

Hong Li[1], Zheng Ma[1], Lijuan Jia[1], Yanmin Li[1], Chunlin Xu[1], Taian Wang[1], Ruili Han[1,2,3], Ruirui Jiang[1,2,3], Zhuanjian Li[1,2,3], Guirong Sun[1,2,3], Xiangtao Kang[1,2,3,*], Xiaojun Liu[1,2,3,*]

[1]College of Animal Science and Veterinary Medicine, Henan Agricultural University, Zhengzhou 450002, China

[2]Henan Innovative Engineering Research Center of Poultry Germplasm Resource, Zhengzhou 450002, China

[3]International Joint Research Laboratory for Poultry Breeding of Henan, Zhengzhou 450002, China

*Corresponding authors

XK: xtkang2001@263.net

XL: xjliu2008@hotmail.com

Supplemental Figure legend

Figure S1 The sequence saturation analysis of six libraries.

A: Sequence saturation analysis of L20-1; **B:** Sequence saturation analysis of L20-2;

C; Sequence saturation analysis of L20-3; **D:** Sequence saturation analysis of L30-1;

E: Sequence saturation analysis of L30-2; **F:** Sequence saturation analysis of L30-3.

Figure S1

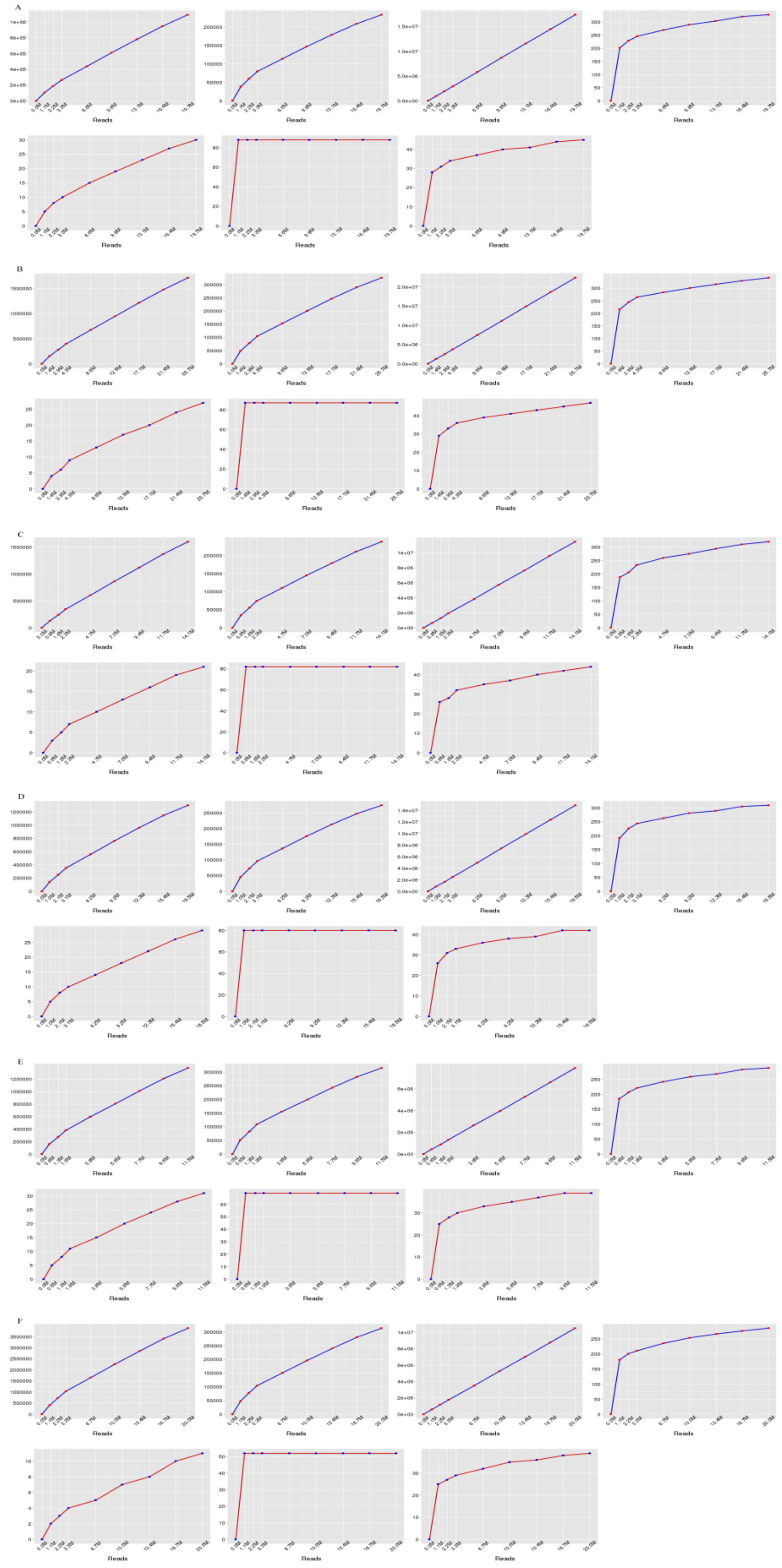

Supplement: Supplementary Information [file srep31766-s1.pdf]
